# Effects of backward-directed resistance on propulsive force generation during split-belt treadmill walking in non-impaired individuals

**DOI:** 10.3389/fnhum.2023.1214967

**Published:** 2023-12-04

**Authors:** Negar Moradian, Mansoo Ko, Christopher P. Hurt, David A. Brown

**Affiliations:** ^1^Department of Physical Therapy, School of Health Professions, The University of Texas Medical Branch at Galveston, Galveston, TX, United States; ^2^Department of Physical Therapy, School of Health Professions, The University of Alabama at Birmingham, Birmingham, AL, United States

**Keywords:** walking, propulsion, split-belt treadmill, ground reaction force, backward-directed resistance

## Abstract

**Introduction:**

Backward-directed resistance is the resistance applied in the opposite direction of the individual’s walking motion. Progressive application of backward-directed resistance during walking at a target speed engages adaptive motor control to maintain that speed. During split-belt walking, a motor control strategy must be applied that allows the person to keep up with the two belts to maintain their position on the treadmill. This situation becomes more challenging when progressive resistance is applied since each limb needs to adapt to the greater resistance to maintain the position. We propose that strategies aimed at changing relative propulsion forces with each limb may explain the motor control strategy used. This study aimed to identify the changes in propulsive force dynamics that allow individuals to maintain their position while walking on an instrumented split-belt treadmill with progressively increasing backward-directed resistance.

**Methods:**

We utilized an instrumented split-belt treadmill while users had to overcome a set of increasing backward-directed resistance through the center of mass. Eighteen non-impaired participants (mean age = 25.2 ± 2.51) walked against five levels of backward resistance (0, 5, 10, 15, and 20% of participant’s body weight) in two different modalities: single-belt vs. split-belt treadmill. On the single-belt mode, the treadmill’s pace was the participant’s comfortable walking speed (CWS). In split-belt mode, the dominant limb’s belt pace was half of the CWS, and the non-dominant limb’s belt speed was at the CWS.

**Results:**

We assessed differences between single-belt vs. split-belt conditions in the slope of the linear relationship between change in propulsive impulse relative to change of backward resistance amount. In split-belt conditions, the slower limb showed a significantly steeper increase in propulsion generation compared to the fast limb across resistance levels.

**Discussion:**

As a possible explanation, the slow limb also exhibited a significantly increased slope of the change in trailing limb angle (TLA), which was strongly correlated to the propulsive impulse slope values. We conclude that the motor control strategy used to maintain position on a split-belt treadmill when challenged with backward-directed resistance is to increase the propulsive forces of the slow limb relative to the fast limb by progressively increasing the TLA.

**Clinical trial registration:**

ClinicalTrials.gov, identifier NCT04877249.

## 1 Background

Walking is a complicated motor activity requiring the generation of lower-limb muscular forces that provide support and propel the body step-by-step (Lacquaniti et al., 2012). These propulsion forces establish a walking speed, which is a valid, reliable, and sensitive measure that can assess and monitor functional status and overall health in various populations (Middleton et al., 2015). Walking speed is regulated biomechanically by the timing and magnitude of the anterior component of the ground reaction force, i.e., the propulsive force (Kuhman and Hurt, 2019). At the end of the stance phase, the trailing limb generates vertical and horizontal forces that accelerate (Ellis et al., 2014) and redirect the body’s center of mass (COM) forward and upward during push-off (Kuo and Donelan, 2010; Kuhman and Hurt, 2019). At the same time, trailing limb forces are countered by lead limb foot strike, which generates a braking force that transitions the center of mass from one inverted pendular arc to the next (Hsiao et al., 2016b; Pimentel et al., 2022). During steady-speed walking, propulsion, and braking force are relatively balanced during the step-to-step transition, and their interaction explains much of the acceleration and deceleration in walking speed within and between steps (Peterson et al., 2011).

Considering the importance of propulsion force in regulating the walking speed, and since it is generated in the anterior-posterior direction, some studies have explored the effect of different levels of backward-directed resistance, the force applied in the opposite direction of the individual’s walking motion, applied at the COM while walking at constant speed (Gottschall and Kram, 2003; McGowan et al., 2009; Naidu et al., 2019) or in an uphill environment (McGowan et al., 2008; Gottschall and Nichols, 2011; Franz and Kram, 2012; Janshen et al., 2017) on propulsion generation. They demonstrated by adding backward resistance or increasing the inclination level, the amount of propulsion generated by each limb increases. Among these, some studies have focused on how walking at different steady-state speeds affects propulsion generation and demonstrated that the faster the walking speed, the greater the generation of propulsive force (Nilsson and Thorstensson, 1989; Sousa and Tavares, 2012; Hsiao et al., 2016b). Because propulsion demands were fixed, these studies primarily focused on the effect of speed and treadmill inclination on propulsion generation for both limbs and did not investigate the relative amount of each limb’s contribution. However, many orthopedic and neurologic conditions result in individuals walking with an asymmetrical gait pattern. Over the last few decades, various split-belt treadmill paradigms have revealed how walking on two independently moving treadmill belts results in discrete and instantaneous spatiotemporal interlimb and intralimb parameter changes with distinctive adaptation and post-adaptation aftereffects (Helm and Reisman, 2015; Buurke et al., 2018; Oshima et al., 2021; Kuhman et al., 2022). Due to the strongly coupled nature of bipedal walking, it’s difficult to target one limb propulsion generation without affecting the other limb during walking using split-belt speeds alone.

With asymmetrical gait patterns, one of the limbs may be weaker and thus not contribute equally to propulsion. In these cases, we need to explore the basic neuromechanical conditions and strategies that can encourage the weaker limb to increase its contribution to the total propulsion that is required to maintain the speed and position on the treadmill. In Hurt et al. (2022) performed a split-belt study on three different levels of inclinations, 0°, 5° and 10° degrees, with healthy participants. In this study, they demonstrated that regardless of inclination, positive ankle work on the fast belt is always higher in comparison with the slow limb. However, they did not evaluate the posterior ground reaction force (GRF) and propulsive impulse, which are shown to be useful predictors of gait performance in post-stroke gait (Roelker et al., 2019), and only evaluated a narrow range of trials. In addition to that, the inclination was not normalized with individual’s body weight, which might have increased variability in their outcome measures.

With this exploratory study, we explored ways in which the non-impaired nervous system can control and maintain the same target speed on a split-belt treadmill under progressively increasing levels of backward-directed resistance, which is normalized by body weight, to evaluate if the addition of resistance will require more propulsive contribution from the slower or, the faster-moving limb. We hypothesize that the motor control strategy that people use to maintain their position on a split-belt treadmill when challenged with progressively increased backward-directed resistance is to increase the propulsive forces of the slow limb relative to the fast limb. Additionally, we conducted measurements of spatiotemporal parameters and TLA to assess whether these variables offer insights into the heightened propulsion generation observed in response to the resistance challenge. If either limb demonstrates a greater relative propulsive impulse contribution, this result may indicate a possible therapeutic approach to test with individuals with post-stroke hemiparesis. We also identified any braking force mechanics and spatiotemporal gait variables that might be related to any difference in propulsion observed between the fast vs. the slow limb.

## 2 Methods

### 2.1 Participants

Eighteen age-similar healthy young adults (mean = 25.2 years SD = ± 2.51, mean height = 169.24 cm SD = ± 13.83, mean body weight = 70.99 kg SD = ± 10.79, 12 female and 6 male, 13 right limb dominant and 5 left limb dominant) participated in this study after providing informed consent. We obtained Institutional Review Board (IRB) approval from the University of Texas Medical Branch Institutional Review Board. Individuals were excluded if they had orthopedic or neurologic concerns or had experienced unexplained falls. We also excluded participants with a history of uncontrolled cardiac, muscular, or neurological comorbid conditions that might have interfered with the ability to perform mild to moderate physical activity. We performed tests to establish limb dominance, i.e., which limb would they use to kick a ball and which limb would they prefer to stand on for a single limb stance. We dropped one participant due to technical difficulties (the treadmill force plate system was not operating); thus, our data analysis included seventeen participants.

### 2.2 Split-belt robotic treadmill interface

**FIGURE 1:**
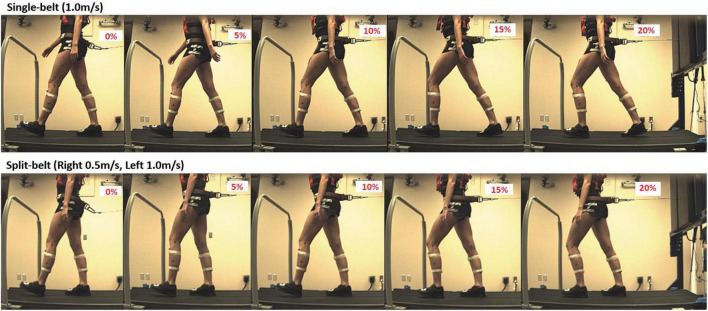
Participants walked in the instrumented double-belt treadmill on single-belt/split-belt conditions. The participants performed walking trials under split and single-belt configurations with five different levels of backward resistance. We set the backward resistance at 0, 5, 10, 15, and 20% of the participant’s body weight. The horizontal backward-directed resistance was applied at the center of mass (COM) using an apparatus built at the back of the treadmill.

A conventional gait marker set was recorded using an eight-Vicon Vantage camera system (Vicon Motion Systems Inc, Denver, CO, USA) to capture bilateral 3D lower body kinematics. Simultaneously, bilateral ground reaction forces and the center of pressure under each foot were collected to synchronize with 3D kinematic data while participants walked on an instrumented split-belt treadmill (Bertec Corp., Columbus, OH, USA). For all trials, participants walked on the instrumented split-belt treadmill with a front safety bar while wearing a safety harness that provided no mechanical support nor impeded movement and was only engaged in the event of a fall or loss of balance. Participants were instructed to use the handrail only in the event of balance disturbance with light touch (Figure 1).

### 2.3 Differential backward resistance

**FIGURE 2:**
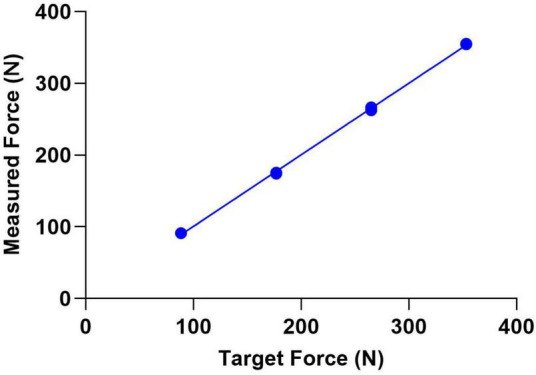
The average measured force of an individual walking in the treadmill environment compared to the targeted resistive force (slope:0.99, *R*^2^:0.99).

We applied backward resistive forces using a resistive force controller apparatus built on the back of our split-belt instrumented treadmill. The application of force was transmitted through a braided metal cable whose height can be adjusted vertically to ensure that the resistive force is applied at the level of the center of mass for individuals. The cable is connected to a constant torque motor and attached to the user via a harness. A fall harness, back padding, and emergency stops provide safety to the participants while they are using the treadmill. The controls for the application of force were modeled as a closed-loop viscoelastic feedback system. For operation, a controller commands the motor via servo control to apply a specified force to the cable. The user works against the applied force by locomoting on the treadmill belt. A force sensor is positioned in line with the cable records and provides feedback on the actual force between the motor and the user (Figure 2). A Kollmorgen AKM43H motor with an AKD servo drive (Kollmorgen, Radford, VA, USA) was chosen to apply the backward-directed resistive force as commanded by the controller.

For this study, we used the resistive force controller to create a resistive force walking condition to match 0, 5, 10, 15, and 20% of the participant’s body weight.

### 2.4 Experimental protocol

All participants selected their CWS inside the interface single-belt mode while we started everyone with the belt speed at 1.0 m/s. Then, we asked participants if they felt comfortable with the current speed, and if it was too fast or too slow, we decreased or increased the belt speed by 0.1 m/s until they confirmed they felt comfortable with the treadmill belt speed. The range of CWS for participants in this study was 0.9- 1.2 m/s. After that, we familiarized participants with the split-belt condition for 1 min, with their dominant limb moving with half of the CWS and non-dominant limb moving at CWS, which was the same configuration for all split-belt trials in this study. The participants performed walking trials under split and single belt configurations with five different levels of backward resistance. We set the backward resistance at 0, 5, 10, 15, and 20% of the participant’s body weight.

We performed the belt speed and backward resistance configuration in the following order: (1) Single-belt with 0% backward resistance, (2) Split-belt with 0% backward resistance, (3) Single-belt with 5% backward resistance, (4) Split-belt with 5% backward resistance, (5) Single-belt with 10% backward resistance, (6) Split-belt with 10% backward resistance, (7) Single-belt with 15% backward resistance, (8) Split-belt with 15% backward resistance, (9) Single-belt with 20% backward resistance, and (10) Split-belt with 20% backward resistance. To avoid fatigue, each trial took place for 30 s, and brief periods of rest were provided between trials.

### 2.5 Data acquisition and processing

The ground reaction force data were collected at 2000 Hz (Bertec Corp., Columbus, OH, USA), and 3D lower body kinematic data were collected with a 20 reflective marker set at 100 Hz (Vicon Motion Systems Inc, Denver, CO, USA). A Woltring digital filter was used to smooth trajectories for computing kinetic data in Vicon Nexus software. Kinetic gait events (foot on and off with a threshold of 15N) per limb were detected from Bertec force plates via MATLAB (version 9.10.). Ground reaction force and segment kinematic data were low-pass filtered with a fourth-order, zero-lag digital Butterworth filter (6 Hz cutoff frequencies) using Visual 3D (C-Motion Inc, Germantown, MD, USA). For each 30-s walking trial on the treadmill, all kinetic and kinematic data were averaged over the gait cycle for each lower limb. Thus, mean impulses are generated by averaging negative or positive horizontal GRFs over gait cycles for 30 s.

Braking impulses were computed as a negative period of time integral of the negative horizontal GRF and normalized to body weight (BW) (Nilsson and Thorstensson, 1989).


$$\int_{h\,s}^{t\,o}\left(-G\,R\,F_{h}\,d\,t\right)*\frac{1}{B\,W}$$

Propulsive impulse was assessed as a positive period of time integral to the positive horizontal GRF and normalized to body weight (Nilsson and Thorstensson, 1989).


$$\int_{h\,s}^{t\,o}\left(G\,R\,F_{h}\,d\,t\right)*\frac{1}{B\,W}$$

Trailing limb angle (TLA) was assessed as the angle between the laboratory’s vertical axis and vector connecting the greater trochanter to the location of the center of pressure at toe-off (Hsiao et al., 2016a).

We calculated the gait cycle duration (i.e., the time between a foot’s two consecutive heel strikes), the stance time (i.e., the time between a foot’s heel strike to toe-off), and the stride length (i.e., the distance between a foot’s two successive heel strike), by multiplying the belt’s speed by stance time.

### 2.6 Statistical analyses

For all statistical analyses, we employed Graphpad Prism (version 9.4.0) and Excel (version 2302). To ensure the normality of our data, we conducted the Shapiro-Wilk normality test on both primary and secondary dependent measures within the single-belt treadmill condition with 0% of body weight applied as backward-directed resistance, yielding a significance level of *p* < 0.05.

To test whether the applied force generated progressively greater horizontal GRF at 0, 5, 10, 15, and 20% BW, we performed a single variant ANOVA test followed by Tukey’s multiple comparisons with an adjusted *p* < 0.05.

For testing propulsive impulse and TLA, we used one sample *t*-test to check if the slopes for each condition (single and split for both limbs, using a Bonferroni’s correction to account for the multiple tests) were greater than zero. Then, we ran a paired sample *t*-test to detect if there was a meaningful difference between fast and slow limbs on the split-belt condition.

In order to test braking impulse, since the data was not linear, we used the log10 for braking impulse values and measured the slope after adding 10^–5^ to all braking data (in order to deal with net zero value) using one-sample *t*-tests to see if it’s different from zero. We then used a paired sample *t*-test to detect if there was a meaningful difference between fast and slow limbs during the split-belt condition.

For testing the relationship between spatiotemporal variables and changes in propulsive impulse, the change in stance time, gait cycle time, and stride length with respect to the increasing backward resistance levels applied (0, 5, 10, 15, and 20% of BW), we used one-sample *t*-tests to detect if slopes were significantly greater than zero. The significance level for all tests was set at an alpha of <0.05.

## 3 Results

### 3.1 Effectiveness of the puller system in generating backward-directed resistance

The puller system successfully generated backward-directed resistive forces against which participants responded with increased propulsive forces. The device produced fairly accurate and repeatable force outputs in the static tests, which were recorded at a frequency of 4 Hz. Errors were less than 10%. For a single figure, we show that the prescribed force very closely matched the force measured by the load sensors in the motor. The regression line was very close to the line of identity (*y* = 0.997, *R*^2^ = 0.99, linear regression) (Figure 2).

**FIGURE 3:**
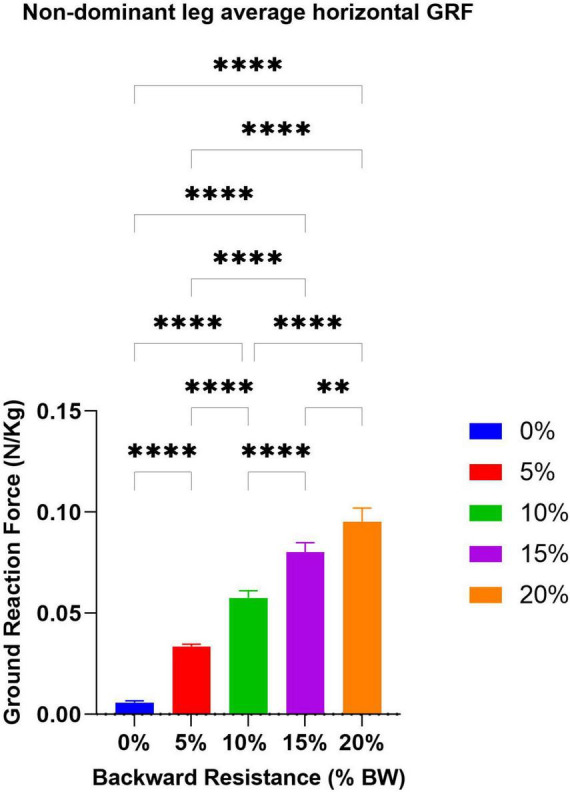
By adding backward-directed resistance (0, 5, 10, 15, and 20% of BW), participants increased their horizontal GRF both on single-belt and split-belt conditions. As shown above, this ANOVA analysis indicates that each level of resistance resulted in a significant increase in net horizontal GRF, thereby confirming that the study was conducted under the specified conditions as explained. **p = 0.005, ****p = 0.001.

Due to the increased resistance provided by the puller system, when averaged across all participants, they increased their mean horizontal GRF force in the single-belt conditions as evidenced by a significant repeated measures ANOVA overall *F*-test (*F* = 126.9 *p* < 0.05, df = 4, ANOVA), and significant differences between each level of resistance as tested by using Tukey’s multiple comparisons (Figure 3).

### 3.2 Propulsive and braking impulses generated against increased resistance

#### 3.2.1 Propulsive impulse

**FIGURE 4:**
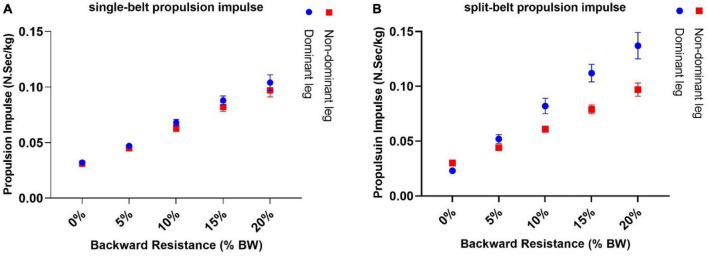
By adding backward directed resistance, the amount of propulsion generation increased both on single-belt **(A)** and the split-belt conditions **(B)**. During the split-belt conditions, the slow limb, which began with a lower propulsive impulse at 0% resistance, increased at a faster rate than the fast limb. As a result, larger propulsive impulse values are observed at higher resistive values.

With additive backward resistance in the single-belt condition, the mean propulsive impulse significantly increased for all participants (Figure 4A). The slopes represent the best-fit line for the change in propulsion over the change in backward resistive force for each participant and are then averaged across all participants. For the dominant limb, slopes on average, were significantly greater than zero [0.0037 ± 0.0003 *NSec*/(*Kg* × %*BW*) *p* < 0.05 after c, one sample *t*-test, Cohen’s *d* = 4], as well as the averaged slopes for the non-dominant limb [0.0034 ± 0.0003 *N* × *Sec*/(*Kg* × %*BW*) *p* < 0.05, one sample *t*-test], but there was no significant difference when comparing the slopes of the two limbs during single-belt conditions (*p* > 0.05, paired sample *t*-test).

However, during the split-belt conditions, the slow limb (dominant limb) initially started with a lower propulsive impulse than the fast limb at 0% resistance (Figure 4B). Then, with higher resistances, the rate of increase was greater for the slow limb compared with the fast limb. Thus, we observed increased propulsive impulse values at higher resistance values. On the split-belt condition, the mean propulsion slope for the dominant limb was significantly greater than zero [0.006 ± 0.001 *N*.*Sec*/(*Kg*.%*BW*) *p* < 0.05, one sample *t*-test], and for the non-dominant limb the slope was also significantly greater than zero [0.003 ± 0.0003 *N*.*Sec*/(*Kg*.%*BW*) *p* < 0.05, one sample *t*-test] and the slow limb had a significantly greater slope than the fast limb (*p* < 0.05, paired sample *t*-test, Cohen’s *d* = 1.5).

#### 3.2.2 Braking impulse

**FIGURE 5:**
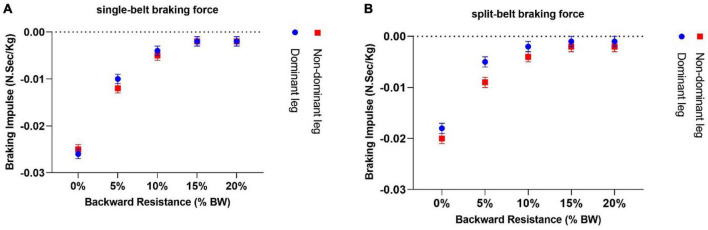
In both single-belt **(A)** and split-belt settings **(B)**, all participants experienced a considerable decrease in braking impulse generation when backward resistance was added. In this graph, we have plotted the original amount of braking impulses; however, for our analysis, since the data was not linear, we used the log10 for braking impulse values and measured the slope after adding 10^–5^.

By adding backward resistance in single-belt conditions, all participants had a significant decline in braking impulse generation (*p* < 0.05, one sample *t*-test) (Figure 5A). The slope represents the best-fit line for the change in braking impulse over the change in backward resistive force. For dominant limb was significantly less than zero [−0.15 ± 0.02 *N*.*Sec*/(*Kg*.%*BW*) *p* < 0.05, one sample *t*-test], also for the non-dominant limb the slope was significantly less than zero [−0.14 ± 0.016 *N*.*Sec*/(*Kg*.%*BW*) *p* < 0.05, using a Bonferroni’s correction to account for the multiple test, one sample *t*-test], but there was no meaningful difference when comparing the dominant and non-dominant limb’s slopes (*p* > 0.05, paired sample *t*-test).

On split-belt conditions, (the slope for the slow; also dominant limb) was significantly less than zero [−0.15 ± 0.02 *N*.*Sec*/(*Kg*.%*BW*) *p* < 0.05, one sample *t*-test], also the slope for the non-dominant limb was significantly less than zero [−0.110 ± 0.014 *N*.*Sec*/(*Kg*.%*BW*) *p* < 0.05, one sample *t*-test]. However, during the split-belt condition, the rate of decline for braking impulse generation was greater for slower limb in comparison with the fast limb, and they had a significant difference (*p* < 0.05, paired sample *t*-test) (Figure 5B).

### 3.3 Trailing limb angle (TLA)

**FIGURE 6:**
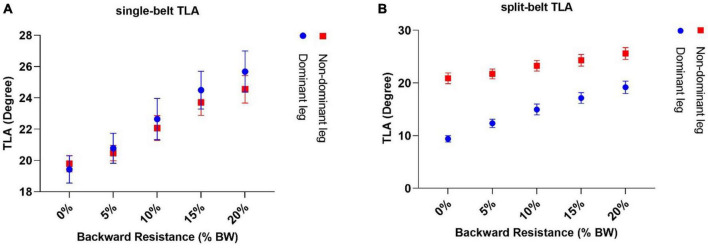
By adding backward-directed resistance, there was a significant slope value greater than zero in TLA both for single-belt **(A)** and split-belt conditions **(B)**. Although the net amount of TLA for the slow limb is lower than the fast limb on the split-belt treadmill, the rate of TLA incrementation was greater for the slow limb than for the fast limb.

On single-belt conditions, the slope represents the best-fit line for change in TLA over change in %BW (Figure 6A). The average TLA slope for the dominant limb was greater than zero (0.32 ± 0.04 /%*BW* *p* < 0.05, using a Bonferroni’s correction to account for the multiple test, one sample *t*-test), also for the non-dominant limb, the average slope was greater than zero (0.24 ± 0.0*4*/%*BW* *p* < 0.05, one sample *t*-test), but there was no significant difference when comparing the dominant and non-dominant limb’s slopes.

On the split-belt conditions, the average TLA slope for the slow limb (dominant limb) was greater than zero (0.49 ± 0.05 /%*BW*, *p* < 0.05, one sample *t*-test). Also, the slope for the non-dominant limb was greater than zero (0.24 ± 0.04 /%*BW* *p* < 0.05, one sample *t*-test) and we observed that the slope value for the slower limb was significantly greater than the faster limb (Figure 6B).

**FIGURE 7:**
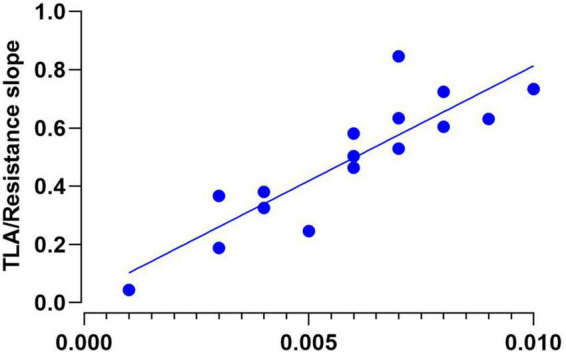
With increasing the backward-directed resistance, we observed a significant correlation between the change in TLA and the change in propulsive impulse (*p* < 0.05, *r* = 0.88, 95% CI = 0.67, 0.95, *R*^2^ = 0.77).

To potentially explain an association between the significant increase in slopes for the slow limb during split-belt conditions, we observed a significant correlation between the TLA slopes compared to the propulsive impulse slopes with increasing backward-directed resistance for the slow limb on the split-belt treadmill (*p* < 0.05, *r* = 0.88, 95% CI = 0.67, 0.95, *R*^2^ = 0.77) (Figure 7). In further support, this association was not observed for the fast limb.

### 3.4 Spatiotemporal variables: stride length, stance time, gait cycle time

In neither single-belt nor split-belt, did we observe any significant changes in mean stride length (mean = 1.1 m, SEM = ± 0.04), mean stance time (mean = 0.73 s, SEM = ± 0.01), and mean gait cycle time (mean = 1.12 s, SEM = ± 0.01) across different levels of horizontal backward resistance (*p* > 0.05, one sample *t*-test).

## 4 Discussion

Using an instrumented split-belt treadmill equipped with a backward resistance controller, we explored the effect of increasing horizontal backward resistance (0, 5, 10, 15, and 20% of body weight) during walking on single-belt vs. split-belt treadmill at the participant’s self-selected CWS. In summary, our results suggest that, during the split-belt conditions, the slow limb, which initially started with a lower propulsive impulse at 0% resistance, demonstrated a greater increase in propulsive impulse compared with the fast limb, resulting in higher propulsive impulse values at higher resistive forces. We also observed that, during the split-belt conditions, the rate of reduction in braking impulse was greater for the slower limb in comparison with the fast limb. Finally, there was a strong association between the increased propulsive impulse with the slow limb and increased TLA.

In agreement with our results, previous studies on resistance and uphill walking at constant speeds, non-impaired individuals scale their peak propulsion forces and duration of propulsion according to the degree of resistance or level of inclination against which they are walking (Lay et al., 2007; Ellis et al., 2014; Naidu et al., 2019). For instance, Naidu et al. (2019) assessed limb propulsion generation in healthy participants against increasing backward-directed resistance at the COM while participants were walking at their CWS in an intent-driven treadmill environment. By conducting this study, they observed a proportional increase in the propulsion generation by incrementing backward resistance without significant changes in vertical limb loading.

Walking on a split-belt treadmill has been presented as an experimental paradigm for exploring the flexibility of neural gait control and rehabilitative training. Tesio et al. (2018) evaluated the ankle work (the main engine of propulsion) on split-belt condition in two sets of speeds, 0.4 vs. 0.8 and 0.8 vs. 1.2 m/s, and showed that the faster limb’s ankle work is 4.8 and 2.2 times higher than the slower side, respectively. In comparison, with a single-belt treadmill with the same speed (slower side speed), ankle work was 1.2 and 1.1 times higher. When viewed along with the results that we present, it appears that ankle work is a significant contributor to speed maintenance during split-belt walking for the fast limb, while TLA, or limb geometry, may be used by the slow limb to keep up with the fast limb.

We aimed to evaluate the propulsive forces while walking against backward-directed resistance in a split-belt paradigm. In a study with a similar aim to ours, Hurt et al. (2022) evaluated the joint or limb work while a split-belt treadmill was combined with uphill walking at 0, 5, and 10° in healthy adult individuals. In this study, they show that the positive mechanical work was greater on the faster limb, regardless of inclination. Our seventeen healthy non-impaired participants demonstrated proportionally increasing propulsion impulse in the slow limb and a greater reduction in braking force in comparison with the fast limb to maintain walking speed in response to increasing backward resistance applied at COM while walking on a split-belt treadmill, without any change in spatiotemporal variables. It is of interest that these two different outcome measures provided different results. Many previous studies have shown that propulsive impulse can distinguish between hemiparetic severity to a greater extent than walking speed (Bowden et al., 2006), as well as kinematic asymmetry (Balasubramanian et al., 2007). We also found that walking against greater backward resistance resulted in greater trailing limb angle, which increased to a greater extent for the slower limb than for the faster limb. According to studies on resistance and uphill walking at constant speeds, healthy, non-impaired individuals modulate their peak propulsion forces and duration of propulsion according to the amount of resistance or level of inclination against which they are walking (McGowan et al., 2009; Franz and Kram, 2014; Naidu et al., 2019). However, none of these studies have evaluated the amount of each limb’s propulsion generation contribution while each limb moves at a distinctly different speed.

Smaller magnitude braking forces at lower levels of resistance in the slower limb can partially explain the reason behind greater propulsion generation at higher resistive forces. Previous work has demonstrated that the limb extension and angle is an important factor that determines the amount of propulsion generation. Peterson et al. (2010) found the position of the foot relative to the body center of mass at the terminal stance is an important predictor of propulsive force for able-bodied individuals and for the non-paretic limb of individuals post-stroke. They proposed that the plantar flexor’s contribution to propulsive force is affected by limb extension. In our study, we observed that as the backward resistance increases, individuals keep up with the speed and resistance by reducing the braking force and putting their slow limb behind their COM so that it has greater extension and is in a better position for propulsion generation.

Regarding the limb angle, participants demonstrated an increase in trailing limb angle in single-belt and split-belt conditions. Although on the split-belt treadmill, the net amount of TLA for the slow limb is lower than the fast limb, the rate of TLA increased to a greater extent for the slow limb compared to the fast limb. We showed that there is a strong correlation between the propulsive impulse generation and TLA angle for the slow limb on the split-belt treadmill. Several studies have demonstrated that an increase in TLA results in increasing propulsion generation (Hsiao et al., 2015a,b; Miyazaki et al., 2019; Naidu et al., 2019). We propose that by using this strategy, participants were able to improve the amount and rate of propulsive-force generation to meet the demands of increased resistance while maintaining their position on the treadmill. These findings are consistent with the previous investigation that has shown that increasing the trailing limb angle correlates with an increase in the amount of propulsion generation required to achieve faster walking speeds (Chen and Lu, 2006; Awad et al., 2014).

This study introduces an innovative approach by integrating split-belt treadmill technology with varying levels of backward-directed resistance to investigate its biomechanical impact on gait for non-impaired individuals. This research aims to explore the potential of this environment to address gait asymmetry and reduce fall risk in individuals with hemiparesis. While the incorporation of backward resistance uniformly enhances propulsion impulse in both limbs, it may exacerbate asymmetry in hemiparetic individuals. Conversely, the use of the split-belt treadmill alone offers limited improvements in propulsion. The combination of these modalities exhibits promise in effectively mitigating gait asymmetry; however, further research involving larger hemiparetic populations is necessary before recommending widespread clinical implementation. This study signifies a significant step forward in optimizing rehabilitation for individuals with hemiparesis and lays the groundwork for future research and refinement of these findings.

## 5 Study limitations

In this study, we limited our exploration of walking function against backward-directed resistance on the split-belt treadmill to the participant’s CWS for the fast limb and half CWS for the slow limb. We didn’t explore the effect of resistance on split-belt treadmills with different speeds, which might have revealed interactions between speed and force differentials. It is also important to consider that in our study; individuals completed only 30 s of walking for each condition that precludes adaptation, which is often shown using kinematic measures such as step length asymmetry. However, it should be noted that Hurt et al. (2022) demonstrated that kinetic data does not show the same adaptation as kinematic data over a longer period of time than the present study.

## 6 Conclusion

We successfully demonstrated that walking on a split-belt treadmill against progressively enhancing backward horizontal resistance, applied at the COM, allowed individuals to generate a greater increase in propulsion impulse and a more rapid reduction in braking impulse by their slower limb compared to the faster limb. The experimental environment of the split-belt treadmill enabled us to evaluate how different amounts of backward resistance affect propulsion, braking, and spatiotemporal variables while each limb moves at a different speed. Our results suggest that backward-directed resistance applied in an environment where each foot goes with a distinct speed generates biomechanical differences in the slow limb compared with the fast limb, that is partially explained by the increased change in trailing limb angle that can enable the slow limb to contribute greater propulsion. We suggest that a follow-up study with participants with post-stroke hemiparesis be conducted to evaluate if employing backward horizontal resistance on a split-belt treadmill can help with improving the paretic limb’s contribution to propulsion generation. If our results find support in a study involving hemiparetic individuals, this environment could receive further examination regarding its therapeutic effectiveness and potential implementation in a clinical setting.

## Data availability statement

The raw data supporting the conclusions of this article will be made available by the authors, without undue reservation.

## Ethics statement

The studies involving humans were approved by the UTMB IRB Revision Board, Galveston, Texas. The studies were conducted in accordance with the local legislation and institutional requirements. The participants provided their written informed consent to participate in this study.

## Author contributions

NM: data collection, data processing, writing–original draft, writing–review and editing, and data analysis. MK: data collection, data processing, and writing–review and editing. CH and DB: data interpretation, writing–review and editing, and funding acquisition. All authors contributed to the article and approved the submitted version.
